# Exploring exposure to multiple psychosocial work factors: prospective associations with depression and sickness absence

**DOI:** 10.1093/eurpub/ckad118

**Published:** 2023-08-02

**Authors:** Jonas Christian Lunen, Reiner Rugulies, Jeppe K Sørensen, Lars L Andersen, Thomas Clausen

**Affiliations:** Department of Psychosocial Work Environment, National Research Centre for the Working Environment, Copenhagen, Denmark; Department of Occupational Health and Social Medicine, Holbæk Hospital, Holbæk, Denmark; Department of Psychosocial Work Environment, National Research Centre for the Working Environment, Copenhagen, Denmark; Section of Epidemiology, Department of Public Health, University of Copenhagen, Copenhagen, Denmark; Department of Psychosocial Work Environment, National Research Centre for the Working Environment, Copenhagen, Denmark; Department of Musculoskeletal Disorders, National Research Centre for the Working Environment, Copenhagen, Denmark; Department of Psychosocial Work Environment, National Research Centre for the Working Environment, Copenhagen, Denmark

## Abstract

**Background:**

Most studies on the psychosocial working environment have focused on evaluating the isolated effect of individual psychosocial work factors or looked at effects through a lens of theories such as job strain or effort–reward imbalance. However, to fathom the intricate nature of workers’ experience of occupational strain, there is a need to investigate the combined and cumulative effects of multiple exposures to psychosocial work factors on workers’ health.

**Methods:**

In this prospective cohort study, we created an additive index (range 0–4) on number of baseline exposures to quantitative demands, emotional demands, role conflicts, and workplace bullying. Via logistic regression and Cox regression, we estimated the association between the additive index of psychosocial work factors and depressive disorder and long-term sickness absence (LTSA). We assessed the onset of depressive disorder using the Major Depression Inventory at 6-month follow-up and the onset of LTSA using a national register during 12-month follow-up.

**Results:**

For onset of depressive disorder, high exposure to any one [odds ratio (OR) 2.98], two (OR 3.14), three (OR 6.44) and all four (OR 9.62) adverse psychosocial work factors predicted a statistically significant increased risk. For onset of LTSA, high exposure to any one [hazard ratio (HR) 1.13], two (HR 1.67), three (HR 2.31) and all four (HR 4.04) psychosocial work factors predicted an increased risk. The two latter associations were statistically significant. Trend tests indicated an exposure–response relationship for both outcomes.

**Conclusions:**

Workers reporting exposure to multiple adverse psychosocial work factors had a higher risk of developing depressive disorder and LTSA.

## Additional content

Additional content An author video to accompany this article is available at: https://oup.cloud.panopto.eu/Panopto/Pages/Viewer.aspx?id=b384a9ae-cf88-47d5-a3b7-b04300920ea7.

## Introduction

Many studies have investigated associations between psychosocial working conditions and health-related outcomes.^1^ Findings show that exposure to various adverse psychosocial work factors is associated with an increased risk of stress-related disorders,^2^ coronary heart disease,^3^ long-term sickness absence (LTSA)^4^^,^^5^ and depression or symptoms of depression.^6^^,^^7^

Contemporary theoretical models within the field of psychosocial work environment research^8–10^ describe how co-existing psychosocial factors intertwine to create the work environment experienced by the worker. These models suggest a complex balance between job demands and job resources, in particular job control, and between efforts and rewards—and these models posit that certain types of imbalances increase the risk of the onset of diseases and disorders. In addition, Hobfoll’s conservation of resources (COR) theory^11^^,^^12^ suggests that a worker has a finite amount of resources to deal with exigent work stressors. Thus, when exposed to several demands at the same time the worker’s ability to cope may decrease accordingly, depleting the worker of resources and energy.^11^

In clinical occupational medicine, practitioners are tasked with assessing patients’ exposure to various psychosocial work factors and symptoms related to occupational stress-related disorders. The process of documenting these exposures and symptoms in a medical anamnesis is highly complex, requiring a systematic and structured approach to understand the multiple factors that often occur simultaneously in the work environment. This complexity presents a significant challenge for occupational health specialists, as they must navigate the intricate nature of the psychosocial work environment experienced by workers in contemporary workplaces. To ensure a comprehensive understanding of the impact of joint exposures in the psychosocial work environment, it is essential to consider as many factors as possible in the assessment and evaluation process.

Although an extensive literature has analyzed the health-related consequences of imbalances between job demands and job resources (e.g. job control), and between efforts and rewards in the work place,^1^^,^^3^^,^^6^^,^^13–16^ only a few studies have investigated the consequences of simultaneous exposure to multiple job demands and/or negative acts.^17–20^ Consequently, evidence about the consequences of workers being exposed to multiple job demands and/or negative acts at the same time is limited.

In this study, we contribute to this literature by exploring the consequences of exposure to combinations of two job demands (quantitative demands and emotional demands), one indicator of a hindrance stressor^21^ (role conflicts), and one indicator of negative acts (workplace bullying). The aim of the present study is to explore the prospective associations between combinations of these four well-established and clinically relevant factors in the psychosocial work environment and the onset of two health-related outcomes: a self-reported measure of depressive disorder and a register-based measure of LTSA.

We decided to focus on these four psychosocial work factors as they are considered relevant in medical occupational clinical practice in Denmark, as well as in Danish work authority legislation. The four psychosocial work factors investigated are in line with The Danish Society of Occupational and Environmental Medicine’s national short clinical guideline to follow when evaluating a patient’s psychosocial work environment.^22^ The four psychosocial work factors are also in line with the ‘Executive order on psychosocial work environment’, published in 2020, by the Danish Working Environment Authority,^23^ which is a government agency purposed to safeguard healthy and safe working conditions in Danish work places. The only work environment factor from the executive order that we did not address with our analyses was ‘work-related violence’ because it had a low prevalence in the study raising concerns about statistical power.

By choosing to investigate these four psychosocial work factors, we aim to grasp the complexity missed when evaluating the consequences of exposure to psychosocial work factors in an isolated form. The present article aims to achieve relevance for both the practice of clinical occupational medicine as well as prophylactically oriented workplace authorities.

## Methods

This study is based on data from a prospective cohort study on employed individuals in Denmark.^24^ In 2015, a questionnaire was distributed to 8958 employed individuals of which 4340 responded (response rate: 48.4%). All respondents received a questionnaire after 6 months of follow-up and 2540 responded (response rate 58.5%). The procedures for population sampling and questionnaire interviews as well as potential biases due to non-response at baseline have been described in detail elsewhere.^24^ We merged data from the baseline survey with data from the Danish Register for Evaluation of Marginalization (DREAM),^25^ which is a national register that includes information on all social transfer payments in Denmark, including sickness absence benefits.

In our analyses of the onset of depressive disorder, we used data from the baseline and the 6-month follow-up study (*n* = 2540). We excluded 229 participants with a baseline Major Depression Inventory (MDI) score ≥21, as this would indicate an already existing depressive disorder. We further excluded 153 participants with missing values on main study variables. This yielded a study population of 2158 participants for the analyses using depressive disorder as outcome. In the analyses of the onset of LTSA, we used data from the baseline study that were merged with data from DREAM (*n* = 4340). We excluded 493 participants with LTSA 2 years preceding the baseline survey. We further excluded 333 participants with missing data on the main study variables. This yielded a study population of 3514 participants for the analyses using LTSA as an outcome.

An analysis of nonresponse at baseline showed that women and older respondents were significantly more likely to participate than men and younger respondents.^24^ In an analysis of attrition from baseline to follow-up, we found no significant differences between men and women, while older respondents were significantly more likely to respond at follow-up than younger respondents. Additional attrition analyses showed no statistically significant differences in the prevalence of depressive disorders at baseline or in the incidence of long-term sickness absence during follow-up when comparing participants to non-participants.^26^

### Exposure variables

We deployed measures from the Danish Psychosocial Work Environment Questionnaire (DPQ)^24^ as predictors in the analyses: Quantitative demands (four items; Cronbach’s α: 0.84). Emotional demands (four items; Cronbach’s α: 0.83). Role conflicts (four items; Cronbach’s α: 0.78) (see Supplementary table S1 for an overview of all items and their response categories).

Bullying was measured with one item: *Have you been exposed to bullying in your current job during the last 12 months?* (Yes or no) (see Supplementary table S1 for details).

For the analyses, we constructed mean scores for quantitative demands, emotional demands, and role conflicts, and dichotomized individuals into low and high exposure using median split. Workplace bullying was dichotomized into ‘exposed’ (yes) or non-exposed (no). Finally, an additive index ranging from 0 (low exposure to all psychosocial work factors) to 4 (high exposure to all psychosocial work factors) was created.

### Outcome variables

Depressive disorder was measured with the MDI at baseline, and at 6-month follow-up. The MDI contains 12 items about depressive symptoms experienced within the last 14 days. Response options for each item range from 0 (symptom not present at all) to 5 (symptom present all of the time). Total MDI score ranges from 0 to 50, as for two pairs of items only the higher score is considered.

In accordance with the literature, we used a score *≥*21 as an indication for a probable depressive disorder.^27^^,^^28^ The MDI and its clinical validation are described in detail elsewhere.^27–29^

We measured LTSA by linking the baseline survey data to DREAM using the participants’ social security number. In line with previous Danish studies,^16^^,^^20^ we defined LTSA as any sickness absence duration lasting a minimum of six consecutive weeks during 1 year of follow-up after baseline.

### Covariates

We adjusted the estimates for the following confounders: sex, age, educational attainment, job group, smoking, cohabitation with partner, and cohabitation with children.

### Statistical analysis

We analyzed the prospective associations between the additive index of high exposure to the psychosocial work factors at baseline and the onset of depressive disorder at follow-up using logistic regression models to estimate odds ratios (ORs) and 95% confidence intervals (95% CIs). We adjusted all analyses for the covariates in three steps (see table 3) and we excluded respondents with a depressive disorder (MDI score ≥21) at baseline. We also followed these procedures when analyzing associations between each of the four exposure variables and the onset of depressive disorder. We analyzed these associations using the LOGISTIC procedure in SAS 9.4 (SAS Institute, Cary, NC). To assess the robustness of the results, we conducted a sensitivity analysis excluding respondents with a baseline MDI score ≥15 as we did in a previous article.^30^ Thus, we used for exclusion a baseline score that was slightly lower than the cut-off point for defining a depressive disorder (MDI score ≥21). In the sensitivity analysis, depressive disorder at follow-up was still defined as an MDI score ≥21.

**Table 3:** OR and 95% CI for the association between cumulated self-reported exposure to adverse psychosocial work factors at baseline and onset of depressive disorder after 6-month follow-up (*n* = 2158)

Self-reported exposure to number of psychosocial work factors			Risk of onset of depressive disorder after 6 months of follow-up							
			**Crude model** ^a^		**Model 1** ^b^		**Model 2** ^c^		**Model 3** ^d^	
	At risk	Cases *n*/%	OR	95% CI	OR	95% CI	OR	95% CI	OR	95% CI
0	610	13/2.1	1	Reference	1	Reference	1	Reference	1	Reference
1	617	24/3.9	1.86	0.94–3.69	1.85	0.93–3.68	2.21	1.10–4.44	2.98	1.09–4.44
2	471	25/5.3	2.57	1.30–5.09	2.57	1.30–5.08	3.31	1.63–6.72	3.14	1.54–6.43
3	411	37/9.0	4.54	2.38–8.66	4.57	2.40–8.73	7.04	3.53–14.06	6.44	3.19–13.01
4	49	6/12.2	6.41	2.32–17.69	6.91	2.49–19.17	9.00	3.11–26.00	9.62	3.30–28.01

a Crude model: unadjusted.

b Model 1: adjusted for age and sex.

c Model 2: Model 1 plus job group and educational attainment.

d Model 3: Model 2 plus smoking, cohabitation with partner, and cohabitation with children.

We analyzed the associations between the additive index of high exposure to the psychosocial work factors at baseline and the onset of LTSA during follow-up using Cox regression models to estimate hazard ratios (HR) and 95% CI. We measured LTSA during 12 months of follow-up using calendar time as the underlying time axis. We followed participants from the week they filled in the questionnaire until the first onset of LTSA or censoring due to migration, retirement, death or end of follow-up, whichever came first. Respondents with LTSA within a 24-month period prior to baseline were excluded from the analyses. We adjusted all analyses for the covariates in three steps (see table 4). We also followed these procedures when analyzing associations between each of the four exposure variables and the onset of LTSA. We found by visual inspection that the proportional hazard assumption was satisfied in all analyses. In these analyses, we used the PHREG procedure in SAS 9.4 (SAS Institute, Cary, NC).

**Table 4:** HRs and 95% CIs for the association between cumulated self-reported exposure to adverse psychosocial work factors at baseline and onset of long-term sickness absence during 12-month follow-up (*n* = 3514)

Self-reported exposure to number of psychosocial work factors			Risk of onset of long-term sickness absence during 12 months of follow-up							
			**Crude model** ^a^		**Model 1** ^b^		**Model 2** ^c^		**Model 3** ^d^	
	At risk	Cases *n*/%	HR	95% CI	HR	95% CI	HR	95% CI	HR	95% CI
0	953	29/3.0	1	Reference	1	Reference	1	Reference	1	Reference
1	943	26/2.8	0.90	0.53–1.53	0.88	0.52–1.50	1.04	0.61–1.78	1.13	0.65–1.97
2	829	34/4.1	1.35	0.82–2.21	1.36	0.83–2.23	1.59	0.95–2.63	1.67	0.99–2.83
3	681	38/5.5	1.85	1.14––3.00	1.77	1.09–2.87	2.25	1.34–3.77	2.31	1.35–3.97
4	108	11/10.2	3.52	1.76–7.05	3.24	1.62–6.50	3.66	1.80–7.47	4.04	1.95–8.37

a Crude model: unadjusted.

b Model 1: adjusted for age and sex.

c Model 2: Model 1 plus job group and educational attainment.

d Model 3: Model 2 plus smoking, cohabitation with partner, and cohabitation with children.

The data collection for this study was approved by the Danish Data Protection Agency. Approval from an Ethics Committee is not required for survey-based research in Denmark.

## Results

Table 1 shows descriptive statistics for the main study variables. The mean age was 48.2 and 45.7 years in the depressive disorder and LTSA analytic samples, respectively. The proportion of women was 47.0% and 47.1%. Most participants reported high exposure to zero, one or two psychosocial work factors (21.8–28.3%). High exposure to three psychosocial work factors was slightly lower (19.1% and 19.4%) and high exposure to all four factors was substantially lower (2.3% and 3.1%).

**Table 1:** Characteristics of the two analytic samples at baseline

	Analytic sample for depressive disorder (*n* = 2158)	Analytic sample for long-term sickness absence (*n* = 3514)
Age, mean (SD)	48.2 (11.1)	45.7 (11.5)
Sex, *n* (%)		
Men	1143 (53.0)	1858 (52.9)
Women	1015 (47.0)	1656 (47.1)
Educational level, *n* (%)		
Low (basic schooling)	210 (9.8)	321 (9.2)
Middle-low (upper secondary school and vocational education)	497 (23.1)	909 (26.0)
Middle-high (short and intermediate higher education)	907 (42.2)	1425 (40.8)
High (long higher education)	487 (22.7)	749 (21.5)
Other	49 (2.3)	86 (2.5)
Job group, *n* (%)		
Office workers	149 (6.9)	243 (6.9)
Technical draughtsmen	177 (8.2)	273 (7.8)
Teaching and research staff in universities	145 (6.7)	223 (6.4)
Health care helpers	107 (5.0)	170 (4.8)
Primary school teachers	185 (8.6)	264 (7.5)
Medical doctors	160 (7.4)	232 (6.6)
Mail carriers	122 (5.7)	206 (5.9)
Slaughterhouse workers	132 (6.1)	259 (7.4)
Smith workers	124 (5.8)	214 (6.1)
Engineers (construction)	205 (9.5)	315 (9.0)
Sales assistants in shops	115 (5.3)	238 (6.8)
Private bankers	176 (8.2)	318 (9.1)
Business managers	178 (8.3)	291 (8.3)
Police officers	183 (8.5)	291 (8.3)
Current smoker, *n* (%)	329 (15.6)	586 (17.4)
Cohabitating with partner, *n* (%)	1692 (80.4)	2675 (79.5)
Cohabitating with children, *n* (%)	1036 (49.2)	1725 (51.3)
Self-reported exposure to psychosocial work factors, *n* (%)		
Self-reported exposure to high levels of 0 factors	610 (28.3)	953 (27.1)
Self-reported exposure to high levels of 1 factor	617 (28.6)	943 (26.8)
Self-reported exposure to high levels of 2 factors	471 (21.8)	829 (23.6)
Self-reported exposure to high levels of 3 factors	411 (19.1)	681 (19.4)
Self-reported exposure to high levels of 4 factors	49 (2.3)	108 (3.1)

Of the 2158 participants in the analytic sample for the onset of depressive disorder, 105 (4.9%) were recorded with a depressive disorder at the 6-month follow-up. Of the 3514 participants in the analytic sample for the onset of LTSA, 138 (3.9%) were recorded with an LTSA during the 12-month follow-up.

Table 2 shows that participants reporting high exposure to quantitative demands (OR 2.09; 95% CI 1.36–3.21), emotional demands (OR 2.46; 95% CI 1.54–3.95), role conflicts (OR 2.92; 95% CI 1.89–4.51) and workplace bullying (OR 2.72; 95% CI 1.54–4.81) had an increased risk of onset of depressive disorder at follow-up compared with the low-exposed reference group.

**Table 2:** Risk of onset of depressive disorder and long-term sickness absence for participants reporting exposure to high quantitative demands, high emotional demands, high role conflicts and workplace bullying

	Onset of depressive disorder				Onset of long-term sickness absence			
	*N*	Cases *N* (%)	OR	95% CI	*N*	Cases *N* (%)	HR	95% CI
High quantitative demands								
Unexposed	1209	43 (3.6)	1	Reference	1963	69 (3.5)	1	Reference
Exposed	949	62 (6.5)	2.09	1.36–3.21	1551	69 (4.5)	1.58	1.10–2.27
High emotional demands								
Unexposed	1198	46 (3.8)	1	Reference	1927	62 (3.2)	1	Reference
Exposed	960	59 (6.2)	2.46	1.54–3.95	1587	76 (4.8)	1.71	1.16–2.53
High role conflicts								
Unexposed	1245	35 (2.8)	1	Reference	1894	54 (2.9)	1	Reference
Exposed	913	70 (7.7)	2.92	1.89–4.51	1620	84 (5.2)	1.86	1.29–2.66
Workplace bullying								
Unexposed	1992	87 (4.3)	1	Reference	3196	115 (3.6)	1	Reference
Exposed	166	18 (10.8)	2.72	1.54–4.81	318	23 (7.2)	1.80	1.14–2.85

Note: All analyses are adjusted for sex, age, educational attainment, job group, smoking, cohabitation with partner and cohabitation with children.

Table 2 also shows that participants reporting high exposure to quantitative demands (HR 1.58; 95% CI 1.10–2.27), emotional demands (HR 1.71; 95% CI 1.16–2.53), role conflicts (HR 1.86; 95% CI 1.29–2.66) and workplace bullying (HR 1.80; 95% CI 1.14–2.85) had an increased risk of onset of LTSA during follow-up compared with the low-exposed reference group.

Table 3 shows OR and 95% CI for the association between self-reported high exposure to the number of psychosocial work factors at baseline (0–4) and the onset of depressive disorder during a 6-month follow-up. In the fully adjusted model, compared with the low-exposed reference group, high exposure to any one (OR 2.98; 95% CI 1.09–4.44), any two (OR 3.14; 95% CI 1.54–6.43), any three (OR 6.44; 95% CI 3.19–13.01) and all four (OR 9.62; 95% CI 3.30–28.01) of the psychosocial work factors was associated with an increased risk of depressive disorder. All differences between the high-exposure groups and the low-exposed reference group were statistically significant. A test for trend indicated an exposure–response relationship (*P* for trend: <0.001).

Results from a sensitivity analysis, where we excluded participants with a baseline MDI score ≥15 points, yielded slightly lower but directionally similar estimates compared with the estimates reported in table 3 (see Supplementary table S2).

Table 4 shows HR and 95% CI for the association between self-reported high exposure to the number of psychosocial work factors at baseline (0–4) and onset of LTSA during 12-month follow-up. In the fully adjusted model, compared with the low-exposed reference group, exposure to any one (HR 1.13; 95% CI 0.65–1.97), any two (HR 1.67; 95% CI 0.99–2.83), any three (HR 2.31; 95% CI 1.35–3.97) and all four (HR 4.04; 95% CI 1.95–8.37) of the psychosocial work factors was associated with an increased risk of onset of LTSA. Exposure to three or four factors yielded statistically significant results compared with the reference group. A test for trend indicated an exposure–response relationship (*P* for trend: <0.001).

## Discussion

In the present study, we examined exposure to four psychosocial work factors (high quantitative demands, high emotional demands, high role conflicts and workplace bullying) in a prospective analysis with regard to two outcomes: onset of depressive disorder and onset of LTSA. We found an exposure–response relationship between the number of self-reported exposure to the psychosocial work factors and the risk of both onset of depressive disorder and onset of LTSA. Participants reporting exposure to high levels of all four psychosocial work factors had a 9-fold increased risk of onset of depressive disorder and a 4-fold increased risk of onset of LTSA at follow-up compared with participants reporting low exposure to all four factors.

The results suggest that simultaneous, cumulative exposure to multiple psychosocial work factors increases the risk of onset of depressive disorder and LTSA. These findings are in agreement with previous studies reporting that combinations of psychosocial work factors constitute a risk factor for adverse work- and health-related outcomes.^17–20^ Other studies^31–33^ have also reported that the combination of psychosocial demands and physical demands increases workers’ risk of ill health.

This study adds new knowledge on the effects of *combined* factors in the psychosocial work environment, which is relevant for occupational health specialists and work environment authorities when promoting a holistic approach to healthy psychosocial working conditions. Thus, focusing solely on single factors at the workplace may be insufficient in improving and protecting workers’ health, although it could be a step in the way. By considering combinations of psychosocial factors in the work environment, it is possible to identify potential problems and take steps to address them in order to create a healthier and safer work environment. To our knowledge, this is the first study to report a prospective association between the accumulation of four potentially adverse psychosocial work factors and the risk of depressive disorder and LTSA. This adds to the relatively new focus on *combinations* of job demands that have emerged since the study from van Woerkom et al. from 2015.^17^

According to the COR theory,^12^ workers invest their resources to deal with job demands in the work situation. When workers over extended periods are exposed to high levels of job demands or adverse factors in their work environment, their psychological and/or physiological resources are likely to be depleted. This may further reduce their ability to deal successfully with additional job demands or adverse factors in the work environment. This could ultimately lead to a so-called *loss spiral*, as those who lack resources are at greater risk of additional loss,^12^^,^^17^ which again may lead to adverse health-related outcomes, such as depressive disorder or LTSA. In our data, the observed exposure–response relationship serves to both illustrate and support the concept of a loss spiral from the conservation of resources theory. On the basis of COR theory, it could be speculated that a supportive workplace culture may constitute a resource in the psychosocial work environment that could mitigate the risks associated with exposure to multiple adverse psychosocial work factors.^12^^,^^34^ Further, structural and contextual factors need to be considered in attempts to improve the psychosocial work environment.^35^

Another aspect of this study is its potential relevance for insurance medicine. From 2019 to 2021, The Danish Labour Market Insurance yearly handled between 4001 and 4691 cases of reported work-related mental disorders.^36^ After assessment, about 7% of these cases were ultimately recognized as occupational diseases. The average percentage of all recognized occupational disease cases, in the same 3 years, was about 25%.^36^ This discrepancy suggests that professional consensus and understanding about work-related mental illness is not as developed as in other spheres of occupational medicine, e.g. respiratory illnesses, allergies, cancers, etc. In accordance, the weight of evidence in regard to causality between adverse psychosocial working conditions and mental disease is still being vigorously debated.^7^^,^^37^ As our study is an observational study, we cannot rule out that unmeasured confounding biased the associations, and thus the study design limits confidence in interpreting the associations between the four psychosocial work factors and the two outcomes as causal. On the other hand, the exposure–response association and the remarkably strong estimates in the highest exposure group (OR 9.62 for depressive disorder and HR 4.04 for LTSA) increase confidence in a causal interpretation of the associations, as unmeasured confounding needed to be exceptionally pronounced to attenuate these strong estimates towards unity.

### Limitations and strengths

The study deployed two different outcomes in the analyses, one measured using self-reports and the other measured in a national register. This is a strength of the study. However, common method biases may have inflated the magnitude of the associations in the analyses of depressive disorder, as both exposure and outcome were assessed by self-report. This may be a limitation of the study.^38^ It is, therefore, a strength of the study that we have used a register-based measure of LTSA as an outcome as this eliminates the risk of common method biases^38^ and loss to follow-up. However, DREAM does not contain information on the diagnoses the individual cases of LTSA are based upon.

The analysis of non-response at baseline showed that women and older respondents were more likely to participate than men and younger respondents. This must be taken into account in the interpretation of the results. However, when comparing participants to non-participants at follow-up, the attrition analysis showed no statistically significant differences in the prevalence of depressive disorders at baseline or in the incidence of long-term sickness absence during follow-up.

We measured depressive disorder with the MDI, a self-administered rating scale, and not with an individual clinical interview, the gold standard method for ascertaining depressive disorder.^39^ We felt confident in using the MDI, because the instrument and the cut-off point of *≥*21 have previously been validated in clinical studies.^27^^,^^40^ It is possible, though, that workers reporting exposure to multiple adverse psychosocial work factors at baseline also have reported a high MDI score, close to the cut-off point of ≥21 at baseline, and that these workers needed only little change to surpass the defined threshold. This could result in reverse causation and severely bias our results, which is a particular concern in the analyses of depressive disorder in this study, as time of follow-up was only 6 months. Therefore, we conducted a sensitivity analysis excluding workers with a baseline MDI score *≥*15. These results did not differ notably from those of the main analysis, indicating that the results were not driven by small changes among workers who were close to the threshold for a depressive disorder at baseline.

## Conclusion

In this prospective cohort study, we found that the more adverse psychosocial work factors a worker reported being exposed to, the more likely that worker was to experience onset of depressive disorder and LTSA during follow-up. For both depressive disorder and LTSA alike, we found a clear exposure–response relationship with the number of adverse psychosocial work factors a worker was exposed to at baseline.

To our knowledge, this study is the first that reported a prospective association between combinations of quantitative demands, emotional demands, role conflicts and workplace bullying and risk of depressive disorder and LTSA. The results add to the growing awareness that *combinations* of psychosocial work factors are important when evaluating their effect on worker health. In a practical context, the results are important for occupational health specialists, work environment authorities and workplaces to evaluate, maintain and create a healthy work environment.

## Supplementary Material

ckad118_Supplementary_Data

## Data Availability

The data that this study is based upon may be shared on reasonable request to the corresponding author. To fathom the intricate nature of workers’ experience of occupational strain, there is a call for investigating the combined or cumulative effects of multiple exposures to psychosocial work factors on workers’ health. This prospective study investigated the association between cumulative exposure to four adverse psychosocial work factors and two health-related outcomes: depressive disorder and long-term sickness absence. Workers reporting exposure to more adverse psychosocial work factors had a higher risk of developing depressive disorder and long-term sickness absence. The findings of this study contribute to the emerging awareness that the *combination* of psychosocial work factors should play a crucial role when evaluating and assessing the impact of occupational strain on worker health. Occupational health specialists, work environment authorities and employers should heed these findings in their efforts to evaluate, maintain and create safe and healthy work environments.
