# Supplementary material for: Exploring exposure to multiple psychosocial work factors: prospective associations with depression and sickness absence
Source: Eur J Public Health. 2023 Aug 2;33(5):821–7. doi: 10.1093/eurpub/ckad118 (PMC10567252; doi:10.1093/eurpub/ckad118)
Supplement: ckad118_Supplementary_Data [file ckad118_supplementary_data.zip › ckad118_Supplementary_Data/ejph-2023-02-om-0073-File002.pdf]

Supplementary table 1: Overview of items used to operationalize the four psychosocial work factors in the study

Quantitative demands

1 How often is it the case that you do not have time to complete all your work tasks?
2 How often do you receive unscheduled work tasks that place you under time pressure?
3 How often do you have deadlines that are hard to meet?
4 Do you get behind with your work?

Response options: "Always"; "Often"; "Sometimes"; "Rarely"; "Never/almost never"

Emotional demands

1 Are you placed in emotionally demanding situations at work?
2 As a result of your work, do you come into contact with people who oppose you or are aggressive towards you?
3 Do you have to deal with relationships at work that are emotionally challenging?
4 As a result of your work, do you have contact with people who are in difficult situations (e.g. people affected by a serious illness, accidents, grief, crises or social problems)?

Response options: "Always"; "Often"; "Sometimes"; "Rarely"; "Never/almost never"

Role conflicts

1 Do you have to do things in your work that you feel should be done differently?
2 Are there any conflicting demands in your work?
3 Does your job involve tasks that conflict with your personal values?
4 Do you sometimes have to end a task even though you do not feel you have completed it?

Response options: "To a very large extent"; "To a large extent"; "Somewhat"; "To a small extent"; "To a very small extent"

Workplace bullying

1 Have you been exposed to bullying in your current job during the last 12 months?
(Bullying takes place when a person repeatedly and over an extended period of time is exposed to unpleasant or degrading treatment. For bullying to take place the person who is bullied must find it difficult to defend him- or herself.)

Response options: "Yes, daily or almost daily"; "Yes, weekly"; "Yes, monthly"; "Yes, occasionally"; "No"

Supplementary table 2: Odds ratios (OR) and 95% confidence intervals (95% CI) for the association between cumulated self-reported exposure to adverse psychosocial work factors at baseline and onset of depressive disorder after six months follow-up. Results from a sensitivity analysis excluding individuals with an MDI baseline score ≥15 (n=1,958)

				Risk of onset of depressive disorder after six months of follow-up							
				Crude model[a]		Model 1[b]		Model 2[c]		Model 3[d]	
		At risk	Cases n/%	OR	95% CI	OR	95% CI	OR	95% CI	OR	95% CI
**Self-reported exposure to number of psychosocial work factors**	0	581	10/1.7	1	Reference	1	Reference	1	Reference	1	Reference
	1	575	13/2.3	1.32	0.57 to 3.04	1.32	0.57 to 3.03	1.59	0.68 to 3.72	1.60	0.68 to 3.77
	2	425	15/3.5	2.09	0.93 to 4.70	2.08	0.92 to 4.67	2.69	1.15 to 6.28	2.56	1.07 to 6.10
	3	339	19/5.6	3.39	1.56 to 7.38	3.39	1.55 to 7.39	5.60	2.40 to 13.07	4.83	2.01 to 11.62
	4	38	4/10.5	6.72	2.00 to 22.54	7.09	2.11 to 23.90	8.26	2.32 to 29.42	9.49	2.62 to 34.41

[a] Crude model: Unadjusted

[b] Model 1: Adjusted for age and sex

[c] Model 2: Model 1 plus job group, and educational attainment

[d] Model 3: Model 2 plus smoking, cohabitation with partner, and cohabitation with children
